# Changing patterns of nicotine product use and nicotine dependence among United States high‐school students: The National Youth Tobacco Survey, 2014–2023

**DOI:** 10.1111/add.70120

**Published:** 2025-06-25

**Authors:** Sarah E. Jackson, Jamie Brown, Harry Tattan‐Birch, Martin J. Jarvis

**Affiliations:** ^1^ Department of Behavioural Science and Health University College London London UK; ^2^ SPECTRUM Consortium Edinburgh UK

**Keywords:** adolescents, dependence, e‐cigarettes, nicotine, smoking, tobacco products

## Abstract

**Background and aim:**

Concerns have been raised that e‐cigarettes have created a new generation of people addicted to nicotine. This study aimed to measure changes in the proportion of US high‐school students reporting symptoms of nicotine dependence over the past decade, in the context of changing patterns of nicotine product use.

**Design:**

Repeat cross‐sectional analyses of the 2014–2023 National Youth Tobacco Surveys.

**Setting:**

United States of America.

**Participants:**

107 968 high‐school students (14–18y).

**Measurements:**

Nicotine product use was categorised based on self‐reported past‐30‐day use of cigarettes, other combustible tobacco, smokeless/non‐combustible products and e‐cigarettes. Nicotine dependence was operationalised as (a) strong past‐30‐day cravings to use tobacco and (b) wanting to use nicotine products within 30 minutes of waking.

**Findings:**

Past‐30‐day use of any nicotine product decreased from 24.5% (95% confidence interval = 22.5%–26.6%) to 19.6% (16.8%–22.4%) between 2014 and 2017, increased sharply, reaching 31.4% (29.0%–33.7%) in 2019 (driven by an increase in e‐cigarette use), then fell to the lowest level at 12.5% (10.9%–14.1%) by 2023. The proportion who reported symptoms of nicotine dependence was substantially lower, but followed a similar pattern of changes over time. For example, the proportion reporting strong cravings decreased from 7.8% (6.6%–9.0%) to 5.5% (4.3%–6.7%) between 2014 and 2017, increased to 7.9% (6.8%–9.0%) between 2017 and 2018 and remained stable up to 2020, then fell to the lowest level at 2.5% (1.9%–3.1%) by 2023. While dependence symptoms were consistently highest among cigarette users, they were increasingly prevalent among those using only e‐cigarettes after 2017.

**Conclusions:**

The sharp rise in the prevalence of nicotine product use (in particular, e‐cigarettes) among US high‐school students in the late 2010s was short‐lived and was not accompanied by a sustained increase in the overall population burden of nicotine dependence. By 2023, both nicotine product use and nicotine dependence had reached historic lows. However, dependence symptoms increased over time among those using e‐cigarettes only.

## INTRODUCTION

Youth use of tobacco products is a long‐standing public health concern [1]. Rates of smoking have decreased steadily since the late 1990s among young people in the United States (US) [2, 3]. However, a substantial rise in e‐cigarette use (‘vaping’) among young people since 2017 has seen the overall decline in use of nicotine products reverse [4]. This has caused concern that e‐cigarettes are undoing decades of progress and creating a new generation of people addicted to nicotine [5]. It is important to understand how far this is supported by evidence, to inform policy decisions around the regulation of e‐cigarettes and other nicotine products. In this study, we examine how levels of nicotine dependence are changing among high‐school students in the United States in the context of evolving patterns of tobacco and nicotine product use.

In the United States, youth vaping prevalence increased rapidly between 2017 and 2019. According to the 2019 National Youth Tobacco Survey (NYTS; an annual, cross‐sectional, school‐based survey), 31.2% of high‐school students (grades 9–12; ages 14–18) had used an e‐cigarette in the past 30 days [6], up from 11.7% in 2017 [7]. This coincided with a rapid rise in popularity of Juul e‐cigarette products, which had marketing and a sleek design that particularly appealed to young people [8]. As of 2019, the NYTS data showed no corresponding large rise in the proportion of high‐school students reporting symptoms of nicotine dependence [9]. However, some researchers have raised concerns that e‐cigarette users may not have interpreted questions about dependence on tobacco products as applying to them (despite this being explicitly stated in the survey) [10], which warrants further investigation.

Since 2019, the vaping landscape has changed. There was an outbreak of acute lung injuries among (predominantly young) people using vaping products in 2019 [11], which was initially attributed to nicotine‐containing e‐cigarette use before the cause was identified as inhalation of vitamin E acetate, found in illicit cannabis vaping products, but not in nicotine e‐cigarettes [12]. Following the outbreak, young people were exposed to increased negative news stories about vaping and their harm perceptions of e‐cigarettes worsened [13]. There have also been regulatory changes in the United States, including bans on vaping products sold by Juul, the brand associated with the rapid rise in vaping among young people; state‐ and local‐level bans on flavours and/or on‐line sales; and in some places an outright ban on the sale of all vaping products [14, 15, 16].

Perhaps as a result of these factors, vaping prevalence among young people has fallen substantially over the past few years [4]. NYTS data show the prevalence of past‐30‐day e‐cigarette use among US high‐school students fell by almost two‐thirds between 2019 and 2023, from 27.5% [6] to 10.0% [17]. There was a similar decline in the proportion reporting past‐30‐day use of any nicotine product, from 31.2% [6] to 12.6% [17].

However, newer vaping products that use salt‐based nicotine e‐liquids deliver nicotine more efficiently than older‐generation devices [18, 19], potentially leading to increased dependence among the remaining pool of vapers. Recent evidence from the International Tobacco Control (ITC) Youth Tobacco and Vaping Study shows markers of dependence increased between 2017 and 2022 among 16‐ to 19‐year‐olds in the United States, Canada and England who vape, reaching levels comparable to cigarette dependence among those who smoke [10]. This raises questions as to what has happened to the population burden of nicotine dependence among young people, in the context of decreased prevalence of vaping, but potentially increased dependence among those who vape.

This study used NYTS data collected between 2014 and 2023 to describe changes in nicotine product use among high‐school students and to examine the extent to which symptoms of nicotine dependence changed during this period, overall and within users of different nicotine product categories (e‐cigarettes only, smokeless but no combustibles, combustibles but no cigarettes and cigarettes). We also provided estimates accounting for potential under‐reporting of symptoms of nicotine dependence by students using e‐cigarettes only.

## METHODS

### Design

We used cross‐sectional data from the NYTS. Details of the NYTS methodology have been published in full elsewhere [20]. Briefly, the NYTS was established in 1999 and uses a three‐stage cluster sampling procedure to recruit a nationally representative sample of students in grades 6 to 12. Participants provide data on a range of variables relevant to tobacco use via an anonymous questionnaire (administered in paper‐and‐pencil form up to 2018 and on‐line from 2019 onward).

We analysed data from high‐school students (grades 9–12; age 14–18 years) surveyed between 2014 and 2023, because questions on nicotine dependence were consistently included in each year during this period.

### Measures

#### Nicotine product use

Nicotine product use was defined as any use of the following four categories on at least one of the past 30 days: cigarettes, other combustible tobacco (defined as cigars, cigarillos or little cigars; pipes filled with tobacco; bidis; tobacco in a hookah or waterpipe), smokeless/non‐combustible tobacco (chewing tobacco, snuff or dip; snus; heated tobacco products; nicotine pouches; other oral nicotine products) and e‐cigarettes. The question assessing use of e‐cigarettes did not distinguish between nicotine‐containing and nicotine‐free e‐cigarettes. Use of heated tobacco products was assessed from 2019 and nicotine pouches from 2021. The 2023 survey replaced questions assessing use of dissolvable tobacco products with ones assessing use of oral nicotine products more broadly (defined as lozenges, discs, tablets, gums, dissolvable tobacco products and other products).

#### Nicotine dependence

Nicotine dependence was assessed using measures of craving and time to wanting to first use tobacco products after waking. These questions were generally asked with guidance making it clear to respondents that ‘tobacco products’ included e‐cigarettes (although they do not contain tobacco, e‐cigarettes are regulated as a tobacco product in the United States [21]). For example, in 2023, the questions were prefaced with the following instructions: ‘In answering the next 5 questions, please think about all of the tobacco products that you have used in the past 30 days, including e‐cigarettes, cigarettes, cigars, smokeless tobacco, snus, nicotine pouches, other oral nicotine products, hookahs, heated tobacco products, pipe tobacco, bidis, and roll‐your‐own cigarettes.’ Nonetheless, we include a sensitivity analysis exploring the potential impact of any under‐reporting by participants who used e‐cigarettes only who may have mistakenly thought these questions did not apply to them.

Craving was assessed with the question: ‘During the past 30 days, have you had a strong craving or felt like you really needed to use a tobacco product of any kind?’ Responses were dichotomised as ‘yes’ versus other. In 2014, examples were given at the end of the question: ‘such as smoking a cigarette or cigar, or using chewing tobacco’. No examples were given in subsequent surveys.

Time to wanting to first use tobacco products after waking was assessed with the question: ‘How soon after you wake up do you want to use a tobacco product?’ Response options were: (a) I do not want to use tobacco, (b) within 5 minutes, (c) from 6 to 30 minutes, (d) from more than 30 minutes to 1 hour, (e) after more than 1 hour, but less than 24 hours, (f) I rarely want to use tobacco. Responses were dichotomised as wanting to first use tobacco products within 30 minutes of waking (responses a and b) versus other.

Both craving and time to first use after waking are validated and widely employed measures of nicotine dependence among adults [22].

All questions mentioned within this section were asked to all participants up to 2019, but from 2020 they were only asked to those who reported using one or more products in the past 30 days. For consistency across survey years, we imputed these variables as 0 (i.e. not dependent) for non‐users in each annual survey. Note this deviates from the approach we took in our previous analysis of data collected up to 2019 [9].

### Statistical analysis

Data were analysed using R v.4.4.1. We used the *survey* package to account for the complex sampling design.

We calculated estimates (with 95% CI) of the prevalence of past‐30‐day use of different nicotine products by survey year and of the prevalence of nicotine dependence (indexed by past‐30‐day craving and wanting to first use tobacco products within 30 minutes of waking) in relation to survey year and past‐30‐day product use. We used logistic regression to estimate associations of survey year (reference: 2014) with product use and nicotine dependence, reporting results as ORs with 95% CIs.

To explore the potential impact of underestimation of dependence among participants who reported using e‐cigarettes only (on the basis that they may not have understood that the questions applied to them, given e‐cigarettes do not contain tobacco [10]), we recalculated estimates of the population burden of nicotine dependence assigning those using only e‐cigarettes the same level of dependence as cigarette smokers (the product typically associated with the highest level of dependence [9]). For each symptom of dependence, we recalculated the population burden of nicotine dependence within each survey year as follows (a breakdown of these calculations is shown in Data S1):

1. Calculated the proportion of the observed estimate of the population burden of nicotine dependence attributable to those using e‐cigarettes only: %using ecigarettes only×%reporting symptom of dependence among those using ecigarettes only. (Data S1 provides a worked example of the contribution of each product category to the population burden of nicotine dependence in 2023, to show how this works.)
2. Re‐estimated this proportion assuming e‐cigarettes were as dependence‐forming as cigarettes: %using ecigarettes only×%reporting symptom of dependence among those using cigarettes.
3. Calculated the uplift to be applied to the observed estimate of the population burden of nicotine dependence: result of step2–result of step1.
4. Applied the uplift to the observed estimate of the population burden of nicotine dependence: observed estimate of populationburden of nicotine dependence+result of step3.

We plotted these re‐estimated values alongside observed estimates to provide an upper bound for the estimate of the population burden of nicotine dependence (i.e. the total proportion of high‐school students, including those who reported not having used any product in the past 30 days, experiencing symptoms of nicotine dependence).

To explore annual changes in frequent e‐cigarette use and associated symptoms of dependence, we calculated estimates (with 95% CIs) of the prevalence of e‐cigarette use on ≥20 of the past 30 days and of the prevalence of nicotine dependence among frequent e‐cigarette users, by survey year. We also ran cross‐tabulations of (any and frequent) past‐30‐day e‐cigarette use in relation to lifetime cigarette use in 2023, to provide up‐to‐date information on e‐cigarette use among high‐school students who have never smoked.

These analyses were not pre‐registered and should be considered exploratory.

## RESULTS

The total sample size was 107 968 [mean (SD) number of participants per year = 10 797 (2179)].

### Nicotine product use

**FIGURE 1:**
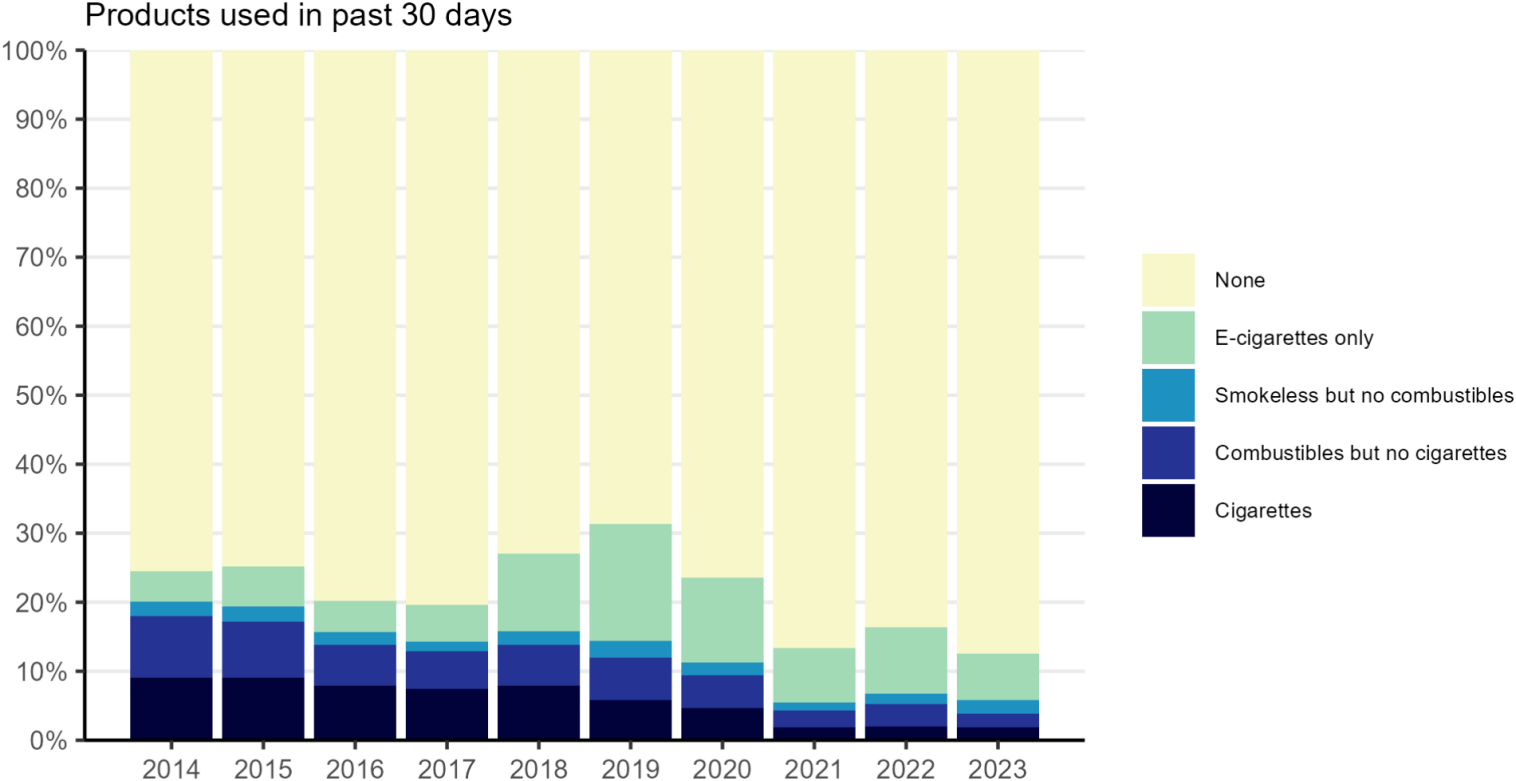
Past‐30‐day nicotine product use among United States high‐school students, 2014 to 2023. Unweighted sample sizes: 2014 *n* = 11 399; 2015 *n* = 9433; 2016 *n* = 10 897; 2017 *n* = 10 186; 2018 *n* = 10 991; 2019 *n* = 10 097; 2020 *n* = 7453; 2021 *n* = 10 515; 2022 *n* = 16 118; 2023 *n* = 10 879. Smokeless includes chewing tobacco, snuff or dip; snus; heated tobacco products; nicotine pouches; other oral nicotine products. Combustibles include cigars, cigarillos or little cigars; pipes filled with tobacco; bidis; tobacco in a hookah or waterpipe. Estimates with 95% CIs are provided in Data S2.

Figure 1 shows patterns of past‐30‐day nicotine product use among high‐school students by survey year. Estimates and ORs with 95% CIs are provided in Data S2.

The prevalence of nicotine product use changed non‐linearly across the period. The proportion of high‐school students who reported having used any nicotine product in the past 30 days decreased between 2014 and 2017, from 24.5% (22.5%–26.6%) to 19.6% (16.8%–22.4%). There was then a sharp increase in prevalence between 2017 and 2019, reaching a high of 31.4% (29.0%–33.7%) in 2019. This trend reversed between 2019 and 2023, with prevalence falling to a new low of 12.5% (10.9%–14.1%) by 2023. The odds of a high‐school student using any nicotine product in the past 30 days were 56% lower in 2023 compared with 2014 [OR = 0.44 (0.37–0.53)].

There were also changes in the types of products being used. The proportion of high‐school students who reported using cigarettes decreased over time, from 9.0% (7.9%–10.3%) in 2014 to 1.8% (1.5%–2.3%) in 2021 and remained at this level in 2023 [1.8% (1.4%–2.4%)]. There was a similar decline in the proportion who reported using other combustible tobacco products (but not cigarettes), from 9.0% (8.1%–10.0%) in 2014 to 2.5% (1.9%–3.1%) in 2021 and 2.0% (1.5%–2.6%) in 2023. As a result, the overall proportion using any combustible tobacco products fell from 18.0% (16.6%–19.4%) in 2014 to 4.3% (3.5%–5.1%) in 2021 and remained relatively stable up to 2023 [3.9% (3.2%–4.5%)]. The proportion who reported using smokeless/non‐combustible products, but no combustibles was consistently low (ranging between 1.2% and 2.4%).

The proportion who reported using e‐cigarettes only was roughly stable between 2014 and 2017 (ranging between 4.4% and 5.8%), then increased rapidly to a high of 17.0% (15.3%–18.7%) in 2019, before falling to 6.7% (5.6%–7.9%) by 2023. Likewise, the proportion who reported frequent (≥20 of the past 30 days) e‐cigarette use was roughly stable between 2014 and 2017 (ranging between 1.9% and 2.4%), increased to a high of 9.3% (7.8%–10.8%) in 2019, then fell to 3.9% (2.8%–5.1%) by 2023 (Data S3). Most [60.7% (55.7%–65.6%)] high‐school students who reported past‐30‐day e‐cigarette use had never smoked a cigarette, but the prevalence of e‐cigarette use (especially frequent use) was greater among those with a history of cigarette use [e.g. the prevalence of frequent past‐30‐day e‐cigarette use was 62.8% (41.4%–80.2%) among those who had smoked at least 100 cigarettes in their lives compared with 2.0% (1.2%–3.2%) among those who had never smoked at all] (Data S4).

Relative to 2014, the odds of a high‐school student in 2023 using e‐cigarettes only in the past 30 days were 55% higher [OR = 1.55 (1.14–2.12)], the odds of using smokeless/non‐combustible products, but no combustibles were similar [OR = 0.93 (0.65–1.33)], and the odds of using cigarettes or other combustible products were approximately 80% lower [OR = 0.19 (0.14–0.25) and OR = 0.21 (0.15–0.28), respectively].

### Nicotine dependence

**FIGURE 2:**
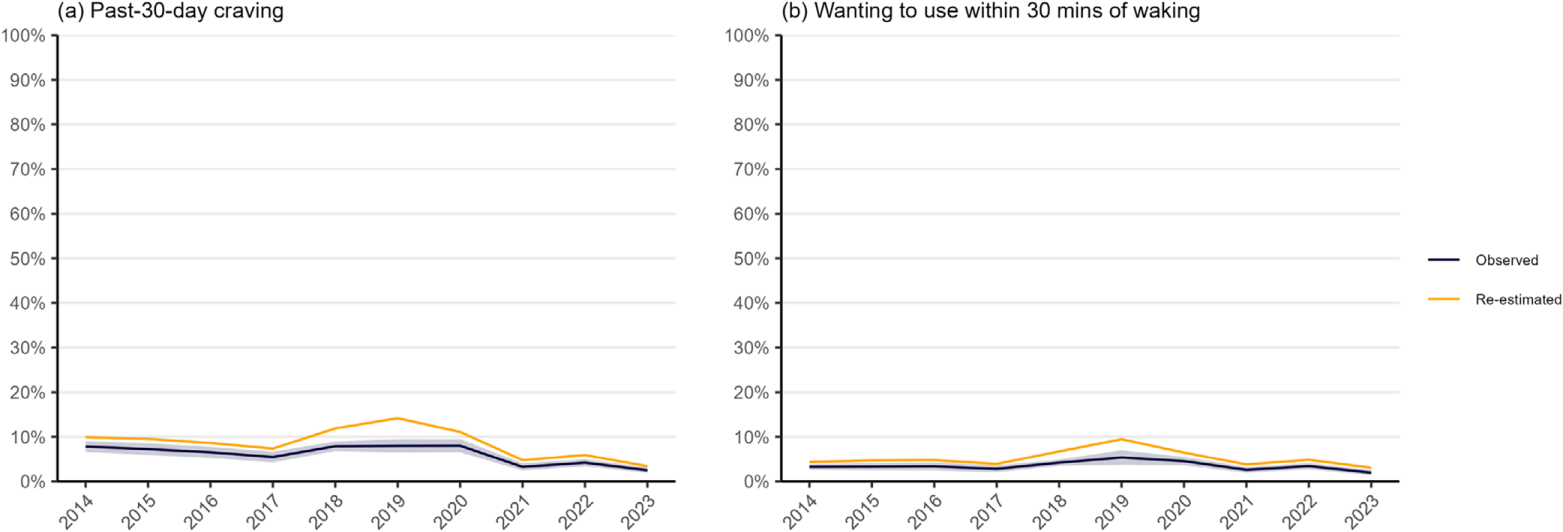
Nicotine dependence among United States high‐school students, 2014 to 2023. Observed values are estimates of the proportion (with 95% CI) who reported symptoms of nicotine dependence among all participants surveyed (including those who did not report any past‐30‐day nicotine product use). Re‐estimated values account for potential underestimation of nicotine dependence among participants who used e‐cigarettes only (on the basis that they may not have thought questions about tobacco products applied to them), assuming that e‐cigarettes were as dependence‐forming as cigarettes (a plausible maximum level of dependence; see Data S1 for details of re‐estimation calculations). Estimates assume no dependence among participants who reported no past‐30‐day nicotine product use. Sample sizes and observed estimates with 95% CIs are provided in Data S5.

**FIGURE 3:**
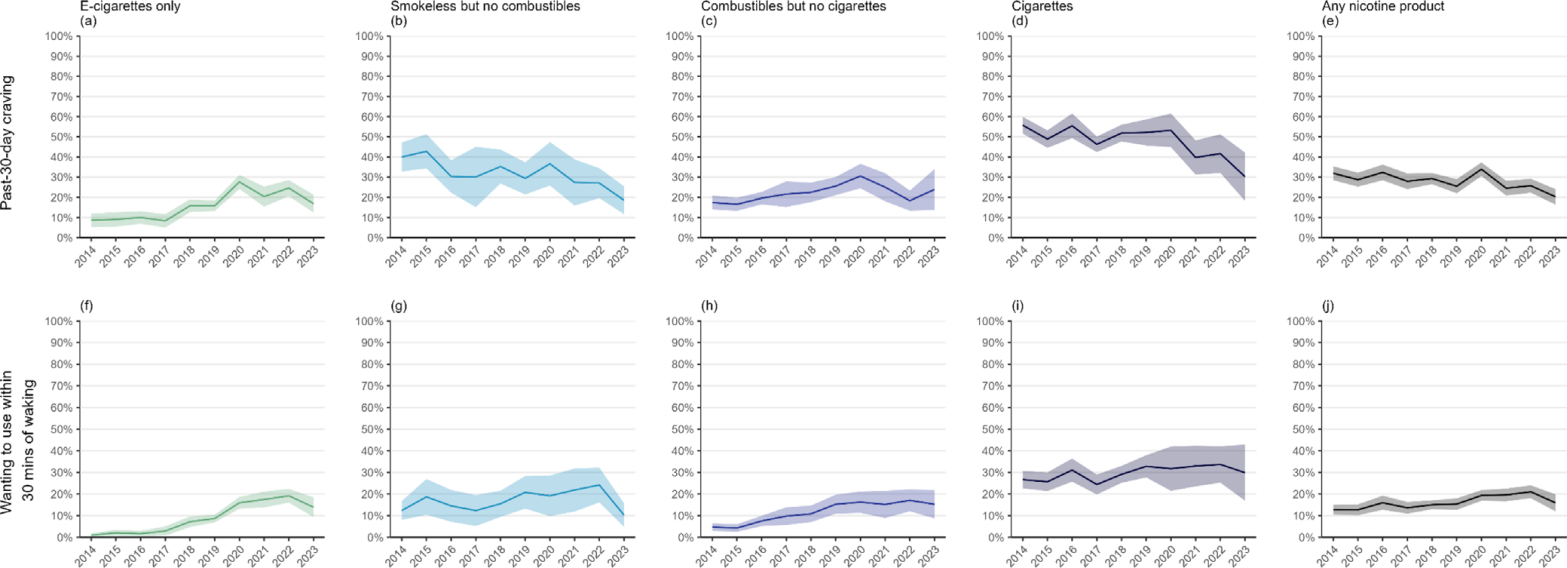
Nicotine dependence among United States high‐school students by product used, 2014 to 2023. Data shown are estimates of the proportions (with 95% CI) of participants using different categories of nicotine products who reported symptoms of nicotine dependence. Sample sizes and estimates with 95% CIs are provided in Data S5.

Figure 2 shows the proportion of all high‐school students reporting symptoms of nicotine dependence by survey year. Figure 3 shows levels of dependence among those using different categories of nicotine products by survey year. Estimates and ORs with 95% CIs are provided in Data S5.

Across the period, the proportion of high‐school students reporting symptoms of nicotine dependence was consistently substantially lower than the proportion who reported past‐30‐day use of any tobacco products. However, changes over time followed a broadly similar pattern. Between 2014 and 2017, the proportion who reported strong craving to use a tobacco product of any kind in the past 30 days decreased from 7.8% (6.6%–9.0%) to 5.5% (4.3%–6.7%). It then increased to 7.9% (6.8%–9.0%) between 2017 and 2018 and remained stable up to 2020, before falling sharply to 3.3% (2.5%–4.1%) in 2021 and then to a new low of 2.5% (1.9%–3.1%) by 2023 [Figure 2(a)]. Relative to 2014, the odds of a high‐school student reporting strong craving to use a tobacco product in the past 30 days were 32% lower in 2017 [OR = 0.68 (0.51–0.90)], similar in 2019 [OR = 1.02 (0.79–1.33)], and 69% lower in 2023 [OR = 0.31 (0.23–0.41)].

Results were similar for the proportion who reported wanting to first use a tobacco product within 30 minutes of waking: little change between 2014 [3.3% (2.6%–3.9%)] and 2017 [2.8% (2.1%–3.6%)], followed by an increase to 5.4% (3.8%–7.0%) by 2019 and subsequent decrease to 2.0% (1.4%–2.6%) by 2023 [Figure 2(b)]. Relative to 2014, the odds of a high‐school student wanting to first use a tobacco product within 30 minutes of waking were similar up to 2017, 67% higher in 2019 [OR = 1.67 (1.15–2.44)], and 40% lower in 2023 than 2014 [OR = 0.60 (0.42–0.85)].

The proportion of high‐school students reporting symptoms of nicotine dependence was consistently highest among those who reported using cigarettes in the past 30 days compared with those who used other combustibles, smokeless/non‐combustible products or e‐cigarettes only (Figure 3). However, there was a notable increase in symptoms of nicotine dependence among those using e‐cigarettes only across the period. The proportion of those who used e‐cigarettes only who reported strong craving to use a tobacco product of any kind in the past 30 days was relatively low and stable (ranging between 8.4% and 10.0%) between 2014 and 2017, increased to 27.6% (24.2%–31.1%) by 2020, and then decreased to 16.9% (12.5%–21.3%) by 2023 [Figure 3(a)]. The proportion who reported wanting to use a tobacco product within 30 minutes of waking was also consistently low (ranging between 1.0% and 2.9%) between 2014 and 2017, then increased steadily to a high of 19.2% (16.0%–22.4%) by 2022. This was followed by an uncertain decrease to 13.9% (9.4%–18.5%) in 2023 [Figure 3(f)]. Patterns were similar among those reporting frequent use of e‐cigarettes (Data S5).

Relative to 2014, the odds of a high‐school student in 2023 reporting strong craving to use a tobacco product in the past 30 days were higher among those who used e‐cigarettes only [OR = 2.15 (1.29–3.57)], but lower among those who used cigarettes [OR = 0.34 (0.19–0.61)] and smokeless/non‐combustible products [OR = 0.34 (0.20–0.58)], with no significant change among those who used other combustibles [OR = 1.48 (0.83–2.66)]. The odds of wanting to use a tobacco product within 30 minutes of waking in 2023 relative to 2014 were higher among those who used e‐cigarettes only [OR = 16.85 (6.17–46.03)] or other (i.e. non‐cigarette) combustibles [OR = 3.67 (1.98–6.80)], but were not significantly different among those who used cigarettes [OR = 1.17 (0.63–2.20)] or smokeless/non‐combustible products [OR = 0.81 (0.41–1.61)].

When we re‐estimated the total population burden of nicotine dependence to account for potential under‐reporting of symptoms among those using e‐cigarettes only (see Data S1 for calculations), changes over time followed a similar pattern, but were more pronounced (Figure 2). Compared with estimates based on the observed data, the peak in 2019 was almost twice as high, reaching 14.2% for past‐30‐day craving and 9.5% for wanting to use within 30 minutes of waking, but values in 2023 were only slightly higher at 3.4% and 3.1%, respectively [compared with observed values of 2.5% (1.9%–3.1%) and 2.0% (1.4%–2.6%)].

## DISCUSSION

In a previous study that analysed NYTS data up to 2019, we concluded that increases in the prevalence of nicotine product use (in particular, e‐cigarettes) among US high‐school students did not appear to have been accompanied by a similar increase in the population burden of nicotine dependence [9]. The present analyses, which include four additional years of data, show that the proportion of high‐school students using any nicotine product peaked in 2019 and has since fallen steeply to a record low in 2023. Use of cigarettes and other combustible products declined steadily between 2014 and 2021 then was stable up to 2023, while e‐cigarette use spiked between 2017 and 2019 before decreasing by 2023. The overall burden of nicotine dependence showed a similar pattern, with a peak in 2019 and a notable reduction by 2023.

Vaping is a contentious issue. Evidence from high‐quality randomised controlled trials shows e‐cigarettes are effective for helping people to stop smoking [23, 24]. This is broadly (although not universally) accepted by researchers, public health organisations and policymakers. However, there is widespread concern that e‐cigarettes are recruiting people into nicotine dependence who would not otherwise have used any nicotine or tobacco [5], and that they have provided a gateway to more harmful cigarette smoking [25]. This has led many countries to impose strict regulations on vaping products [26], which has probably contributed to reduced uptake among young people, but potentially undermined their usefulness for smoking cessation. Our results show no evidence of a gateway effect from vaping to smoking at the population level: despite a sharp rise in e‐cigarette use in 2019, use of cigarettes and other combustible tobacco products continued to fall and in 2023 were lower than ever. Likewise, the spike in e‐cigarette use among high‐school students did not lead to a sustained increase in the population burden of nicotine dependence, which is now at a historically low level.

Although young people who vape are more likely to subsequently initiate smoking (an association found across many longitudinal studies [27]), the available data do not support the presence of a substantial net population‐level gateway effect. If vaping were a major causal driver for smoking uptake, we would expect to see a clear deviation from the long‐term declining trend in youth smoking rates following the period of rapid growth in vaping from 2017 to 2019, even when considering the influence of other tobacco control policies. In contrast, countries such as the United States have experienced declines in youth smoking that began well before the introduction of e‐cigarettes [28], and these trends have continued despite the rise in vaping. This suggests that any individual‐level gateway effect is either minimal or effectively counterbalanced by other interventions. Therefore, although a gateway effect for some individuals is likely, the data indicate that its impact at the population level is too small to be driving overall trends.

We found that while dependence symptoms were consistently highest among cigarette users, they were increasingly prevalent among those using e‐cigarettes only after 2017. This is consistent with data from other surveys [10] and experimental studies [18, 19] showing e‐cigarette devices have become more efficient at delivering nicotine over time—particularly since the introduction of nicotine salts [29]. In our analyses, we explored the possibility that some e‐cigarette users may not have interpreted nicotine dependence questions as applicable to them (even though guidance clarified this). If this were true, it could have led us to underestimate dependence among those using only e‐cigarettes. However, even when we assumed e‐cigarettes were as dependence‐forming as cigarettes, our results did not show a sustained increase in the population burden of dependence. This evidence directly contradicts claims that e‐cigarettes are creating a new generation of people addicted to nicotine [5].

Collectively, our findings show that rises in youth vaping may not necessarily be sustained in the long term. Rates declined sharply following the peak in 2019, much more rapidly than typically seen for smoking. This might mean youth vaping is less difficult to address than smoking, assuming recent product developments (e.g. additions of synthetic coolants) do not make e‐cigarettes more dependence‐forming and difficult to quit. Therefore, a proportionate approach to regulation might aim to reduce the access and appeal of e‐cigarettes to young people (e.g. targeting restrictions at marketing and branding, such as bans on advertising, point‐of‐sale display restrictions or plain packaging), without possibly jeopardising their effectiveness for smoking cessation (e.g. limiting nicotine content). Careful monitoring of trends in youth use and dependence can allow restrictions to be adapted if and when needed.

Finally, we note that prevalence of use of cigarettes and other combustible tobacco products appears to have stalled since 2021 following years of steady decline. Additional policies may be required to address the final approximately 4% of high‐school students smoking combustible tobacco.

This study had several limitations. First, nicotine product use was determined based on self‐reports of any use within the past 30 days. Future studies could offer further insights into changing patterns of dependence by accounting for frequency of use. Second, for surveys conducted from 2020 onward, nicotine dependence questions were only asked to participants who had used a nicotine product in the past 30 days. We coded dependence as 0 (i.e. not dependent) for non‐users, which might underestimate dependence for individuals who use nicotine products infrequently. Third, the survey did not differentiate between nicotine‐containing and nicotine‐free e‐cigarettes, or different types of devices (e.g. older vs. newer salt‐based nicotine e‐cigarettes), which may have varying impacts on dependence. By including use of nicotine‐free e‐cigarettes in our analyses, our results may overestimate the total population burden of nicotine dependence. However, data from other high‐income countries such as the United Kingdom and Canada suggest most young vapers use e‐cigarettes that contain nicotine [30, 31]. Finally, the repeat cross‐sectional design cannot assess causal relationships between changes in (or continued) product use and nicotine dependence over time. Longitudinal data are needed to capture individual trajectories of dependence.

In conclusion, rapid increases in the prevalence of nicotine product use (in particular, e‐cigarettes) among US high‐school students in the late 2010s were short‐lived and were not accompanied by a sustained increase in the population burden of nicotine dependence. By 2023, both nicotine product use and nicotine dependence were at historic low levels. However, dependence symptoms increased over time among those using e‐cigarettes only.

## AUTHOR CONTRIBUTIONS

**Sarah E. Jackson:** Conceptualization (equal); formal analysis (lead); investigation (equal); methodology (equal); visualization (lead); writing—original draft (lead); writing—review and editing (equal). **Jamie Brown:** Conceptualization (supporting); funding acquisition (lead); investigation (equal); methodology (equal); writing—review and editing (equal). **Harry Tattan‐Birch:** Conceptualization (supporting); investigation (equal); methodology (equal); writing—review and editing (equal). **Martin J. Jarvis:** Conceptualization (equal); formal analysis (supporting); investigation (equal); methodology (equal); supervision (lead); writing—review and editing (equal).

## DECLARATION OF INTERESTS

J.B. has received (most recently in 2018) unrestricted research funding from Pfizer and J&J, who manufacture smoking cessation medications. All authors declare no financial links with tobacco companies, e‐cigarette manufacturers or their representatives.

## Supporting information


**Data S1.** Supplementary Material.

**Data S2.** Supplementary Material.

**Data S3.** Supplementary Material.

**Data S4.** Supplementary Material.

**Data S5.** Supplementary Material.

## Data Availability

All data used for these analyses are available on‐line at https://www.cdc.gov/tobacco/about-data/surveys/national-youth-tobacco-survey.html
