# Supplementary material for: Changing patterns of nicotine product use and nicotine dependence among United States high‐school students: The National Youth Tobacco Survey, 2014–2023
Source: Addiction. 2025 Jun 25;120(11):2215–22. doi: 10.1111/add.70120 (PMC12529234; doi:10.1111/add.70120)
Supplement: Supplementary file 1 — Data S1. Supplementary Material. [file ADD-120-2215-s005.docx]

**Table 1.** Re-estimating the population burden of nicotine dependence among US high-school students assuming e-cigarettes are as dependence-forming as cigarettes: past-30-day craving

| **Year** | **% using**  **e-cigarettes only** | **% reporting past-30-day craving among those using e-cigarettes only** | **% reporting past-30-day craving among those using cigarettes** | **% of population reporting past-30-day craving attributable to e-cigarettes only^1^** | **% of population reporting past-30-day craving attributable to e-cigarettes only if they were as dependence-forming as cigarettes^2^** | **Uplift applied to observed estimate of population burden of dependence^3^** |
| --- | --- | --- | --- | --- | --- | --- |
|  |  |  |  |  |  |  |
| 2014 | 4.4% | 8.7% | 55.8% | 0.4% | 2.5% | 2.1% |
| 2015 | 5.8% | 9.1% | 48.9% | 0.5% | 2.8% | 2.3% |
| 2016 | 4.6% | 10.0% | 55.5% | 0.5% | 2.6% | 2.1% |
| 2017 | 5.1% | 8.4% | 46.3% | 0.4% | 2.4% | 1.9% |
| 2018 | 11.2% | 15.9% | 51.8% | 1.8% | 5.8% | 4.0% |
| 2019 | 17.0% | 15.8% | 52.2% | 2.7% | 8.9% | 6.2% |
| 2020 | 12.3% | 27.6% | 53.2% | 3.4% | 6.5% | 3.1% |
| 2021 | 7.9% | 20.3% | 39.7% | 1.6% | 3.1% | 1.5% |
| 2022 | 9.7% | 24.6% | 41.7% | 2.4% | 4.0% | 1.7% |
| 2023 | 6.7% | 16.9% | 30.2% | 1.1% | 2.0% | 0.9% |
|  |  |  |  |  |  |  |

^1^ Estimate based on observed data: % using e-cigarettes only multiplied by % reporting past-30-day craving to use a tobacco product among those using e-cigarettes only.

^2^ Re-estimate based on the assumption that e-cigarettes are as dependence-forming as cigarettes: % using e-cigarettes only multiplied by % reporting past-30-day craving to use a tobacco product among those using cigarettes.

^3^ Amount the estimate of the population burden of nicotine dependence (based on past-30-day craving) needs to be increased by to account for the re-estimation of dependence among those using e-cigarettes only: % of population reporting past-30-day craving attributable to e-cigarettes only if they were as dependence-forming as cigarettes minus % of population reporting past-30-day craving attributable to e-cigarettes only.

**Table 2.** Re-estimating the population burden of nicotine dependence among US high-school students assuming e-cigarettes are as dependence-forming as cigarettes: wanting to use within 30 minutes of waking

| **Year** | **% using**  **e-cigarettes only** | **% reporting wanting to use within 30 mins among those using e-cigarettes only** | **% reporting wanting to use within 30 mins among those using cigarettes** | **% of population reporting wanting to use within 30 mins attributable to e-cigarettes only^1^** | **% of population reporting wanting to use within 30 mins attributable to e-cigarettes only if they were as dependence-forming as cigarettes^2^** | **Uplift applied to observed estimate of population burden of dependence^3^** |
| --- | --- | --- | --- | --- | --- | --- |
|  |  |  |  |  |  |  |
| 2014 | 4.4% | 1.0% | 26.7% | 0.0% | 1.2% | 1.1% |
| 2015 | 5.8% | 2.0% | 25.7% | 0.1% | 1.5% | 1.4% |
| 2016 | 4.6% | 1.7% | 31.1% | 0.1% | 1.4% | 1.4% |
| 2017 | 5.1% | 2.9% | 24.4% | 0.1% | 1.2% | 1.1% |
| 2018 | 11.2% | 7.2% | 29.1% | 0.8% | 3.3% | 2.5% |
| 2019 | 17.0% | 8.7% | 32.8% | 1.5% | 5.6% | 4.1% |
| 2020 | 12.3% | 15.9% | 31.7% | 2.0% | 3.9% | 1.9% |
| 2021 | 7.9% | 17.5% | 33.0% | 1.4% | 2.6% | 1.2% |
| 2022 | 9.7% | 19.2% | 33.7% | 1.9% | 3.3% | 1.4% |
| 2023 | 6.7% | 13.9% | 29.9% | 0.9% | 2.0% | 1.1% |
|  |  |  |  |  |  |  |

^1^ Estimate based on observed data: % using e-cigarettes only multiplied by % reporting wanting to use a tobacco product within 30 minutes of waking among those using e-cigarettes only.

^2^ Re-estimate based on the assumption that e-cigarettes are as dependence-forming as cigarettes: % using e-cigarettes only multiplied by % reporting wanting to use a tobacco product within 30 minutes of waking among those using cigarettes.

^3^ Amount the estimate of the population burden of nicotine dependence (based on wanting to use within 30 minutes of waking) needs to be increased by to account for the re-estimation of dependence among those using e-cigarettes only: % of population reporting wanting to use within 30 minutes attributable to e-cigarettes only if they were as dependence-forming as cigarettes minus % of population reporting wanting to use within 30 minutes attributable to e-cigarettes only.

**Table 3.** Worked example: the contribution of different types of nicotine product use to the total population burden of nicotine dependence among US high-school students in 2023

| **Product use category** | **% using^1^** | **% reporting past-30-day craving among those using^2^** | **% of population reporting past-30-day craving attributable to product use^3^** |
| --- | --- | --- | --- |
|  |  |  |  |
| E-cigarettes only | 6.7% | 16.9% | 1.1% |
| Smokeless but no combustibles | 2.0% | 18.5% | 0.4% |
| Combustibles but no cigarettes | 2.0% | 23.9% | 0.5% |
| Cigarettes | 1.9% | 30.2% | 0.6% |
|  |  |  |  |
| *Any nicotine product* | 12.5% | 2.5% | 2.5% |
|  |  |  |  |

^1^ Statistics from **Supplementary File 2**.

^2^ Statistics from **Supplementary File 3**.

^3^ Calculated as % using x % reporting past-30-day craving among those using.
