# Supplementary material for: Changing patterns of nicotine product use and nicotine dependence among United States high‐school students: The National Youth Tobacco Survey, 2014–2023
Source: Addiction. 2025 Jun 25;120(11):2215–22. doi: 10.1111/add.70120 (PMC12529234; doi:10.1111/add.70120)
Supplement: Supplementary file 2 — Data S2. Supplementary Material. [file ADD-120-2215-s002.docx]

**Table 1.** Past-30-day nicotine product use among US high-school students, 2014 to 2023

|  |  | **% [95% CI] reporting past-30-day use of…** | | | | |
| --- | --- | --- | --- | --- | --- | --- |
| **Year** | ***N*** | **E-cigarettes only** | **Smokeless but no combustibles^1^** | **Combustibles but no cigarettes^2^** | **Cigarettes** | **Any nicotine product** |
|  |  |  |  |  |  |  |
| 2014 | 11,399 | 4.4 [3.4–5.6] | 2.1 [1.6–2.7] | 9.0 [8.1–10.0] | 9.0 [7.9–10.3] | 24.5 [22.5–26.6] |
| 2015 | 9,433 | 5.8 [4.9–7.0] | 2.2 [1.6–3.1] | 8.1 [7.2–9.2] | 9.1 [7.7–10.7] | 25.2 [23.1–27.4] |
| 2016 | 10,897 | 4.6 [3.8–5.5] | 1.8 [1.4–2.4] | 6.0 [5.2–6.8] | 7.8 [6.5–9.4] | 20.2 [18.2–22.2] |
| 2017 | 10,186 | 5.4 [4.2–6.8] | 1.4 [1.0–2.0] | 5.4 [4.6–6.4] | 7.4 [6.1–9.0] | 19.6 [16.8–22.4] |
| 2018 | 10,991 | 11.2 [9.6–13.1] | 1.9 [1.5–2.5] | 5.9 [5.1–6.9] | 7.9 [6.8–9.2] | 27.0 [25.0–28.9] |
| 2019 | 10,097 | 17.0 [15.3–18.7] | 2.4 [1.8–3.2] | 6.2 [5.2–7.3] | 5.8 [4.5–7.4] | 31.4 [29.0–33.7] |
| 2020 | 7,453 | 12.3 [10.9–14.0] | 1.9 [1.4–2.5] | 4.8 [4.1–5.7] | 4.6 [3.5–6.0] | 23.6 [21.0–26.3] |
| 2021 | 10,515 | 7.9 [6.6–9.3] | 1.2 [0.9–1.6] | 2.5 [1.9–3.1] | 1.8 [1.5–2.3] | 13.3 [11.4–15.2] |
| 2022 | 16,118 | 9.7 [8.6–10.9] | 1.5 [1.2–2.0] | 3.2 [2.6–3.9] | 2.0 [1.6–2.5] | 16.4 [14.6–18.3] |
| 2023 | 10,879 | 6.7 [5.6–7.9] | 2.0 [1.5–2.5] | 2.0 [1.5–2.6] | 1.8 [1.4–2.4] | 12.5 [10.9–14.1] |
|  |  |  |  |  |  |  |

CI, confidence interval.

^1^ Includes chewing tobacco, snuff, or dip; snus; heated tobacco products; nicotine pouches; other oral nicotine products.

^2^ Includes cigars, cigarillos, or little cigars; pipes filled with tobacco; bidis; tobacco in a hookah or waterpipe.

**Table 2.** Odds of past-30-day nicotine product use among US high-school students, 2014 to 2023

|  | **OR [95% CI] reporting past-30-day use of…** | | | | |
| --- | --- | --- | --- | --- | --- |
| **Year** | **E-cigarettes only** | **Smokeless but no combustibles^1^** | **Combustibles but no cigarettes^2^** | **Cigarettes** | **Any nicotine product** |
|  |  |  |  |  |  |
| 2014 | Ref | Ref | Ref | Ref | Ref |
| 2015 | 1.35 [1.00–1.82] | 1.04 [0.69–1.58] | 0.90 [0.75–1.07] | 1.00 [0.80–1.26] | 1.04 [0.89–1.21] |
| 2016 | 1.04 [0.76–1.42] | 0.86 [0.59–1.27] | 0.64 [0.54–0.77] | 0.86 [0.67–1.10] | 0.78 [0.66–0.92] |
| 2017 | 1.23 [0.86–1.75] | 0.65 [0.41–1.01] | 0.58 [0.47–0.72] | 0.81 [0.63–1.04] | 0.75 [0.61–0.93] |
| 2018 | 2.73 [2.01–3.72] | 0.92 [0.64–1.33] | 0.64 [0.52–0.78] | 0.87 [0.70–1.08] | 1.14 [0.98–1.32] |
| 2019 | 4.44 [3.35–5.88] | 1.15 [0.78–1.70] | 0.67 [0.54–0.82] | 0.62 [0.46–0.83] | 1.40 [1.20–1.64] |
| 2020 | 3.05 [2.28–4.08] | 0.89 [0.61–1.29] | 0.51 [0.42–0.63] | 0.49 [0.36–0.67] | 0.95 [0.79–1.14] |
| 2021 | 1.85 [1.36–2.52] | 0.56 [0.37–0.85] | 0.25 [0.20–0.33] | 0.19 [0.14–0.25] | 0.47 [0.39–0.58] |
| 2022 | 2.32 [1.74–3.10] | 0.73 [0.51–1.04] | 0.33 [0.26–0.42] | 0.21 [0.16–0.27] | 0.60 [0.51–0.72] |
| 2023 | 1.55 [1.14–2.12] | 0.93 [0.65–1.33] | 0.21 [0.15–0.28] | 0.19 [0.14–0.25] | 0.44 [0.37–0.53] |
|  |  |  |  |  |  |

CI, confidence interval; OR, odds ratio.

Data shown are the odds of product use in each year, relative to 2014. Each column shows results from a separate logistic regression model.

^1^ Includes chewing tobacco, snuff, or dip; snus; heated tobacco products; nicotine pouches; other oral nicotine products.

^2^ Includes cigars, cigarillos, or little cigars; pipes filled with tobacco; bidis; tobacco in a hookah or waterpipe.
