# Supplementary material for: Changing patterns of nicotine product use and nicotine dependence among United States high‐school students: The National Youth Tobacco Survey, 2014–2023
Source: Addiction. 2025 Jun 25;120(11):2215–22. doi: 10.1111/add.70120 (PMC12529234; doi:10.1111/add.70120)
Supplement: Supplementary file 3 — Data S3. Supplementary Material. [file ADD-120-2215-s001.docx]

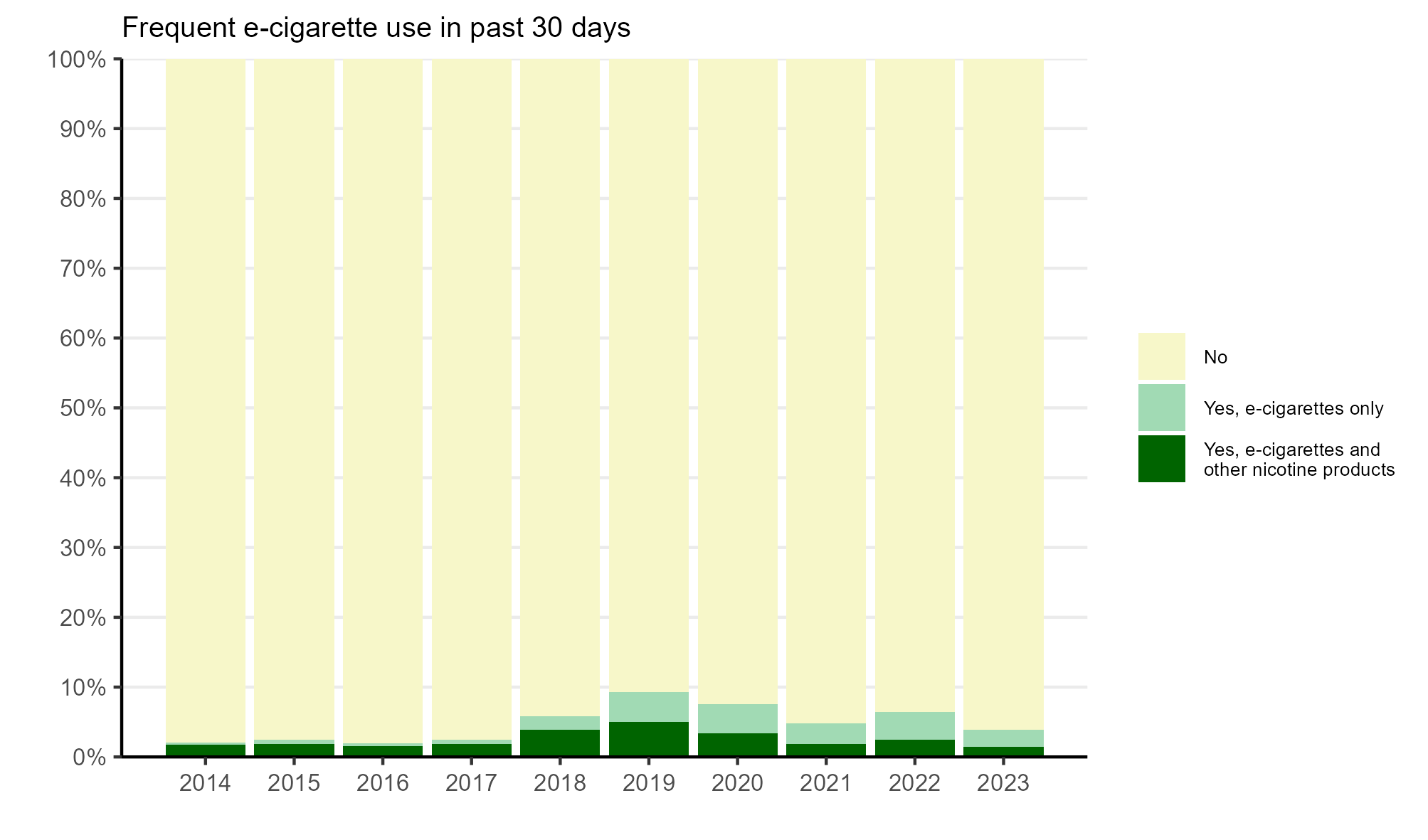


**Figure 1. Frequent e-cigarette use (≥20 of the past 30 days) among US high-school students, 2014 to 2023.** Unweighted sample sizes: 2014 *n*=11,399; 2015 *n*=9,433; 2016 *n*=10,897; 2017 *n*=10,186; 2018 *n*=10,991; 2019 *n*=10,097; 2020 *n*=7,453; 2021 *n*=10,515; 2022 *n*=16,118; 2023 *n*=10,879.

**Table 1.** Frequent e-cigarette use (≥20 of the past 30 days) among US high-school students, 2014 to 2023

|  | **% [95% CI] reporting frequent past-30-day use^1^ of…** | | |
| --- | --- | --- | --- |
| **Year** | **E-cigarettes** | **E-cigarettes only** | **E-cigarettes alongside other nicotine products** |
|  |  |  |  |
| 2014 | 2.1 [1.4–2.7] | 0.3 [0.1–0.5] | 1.8 [1.2–2.3] |
| 2015 | 2.4 [1.8–3.1] | 0.5 [0.3–0.8] | 1.9 [1.4–2.4] |
| 2016 | 1.9 [1.5–2.4] | 0.4 [0.2–0.5] | 1.6 [1.2–1.9] |
| 2017 | 2.4 [1.6–3.2] | 0.6 [0.4–0.8] | 1.8 [1.2–2.5] |
| 2018 | 5.9 [4.9–6.8] | 2.0 [1.4–2.6] | 3.9 [3.2–4.6] |
| 2019 | 9.3 [7.8–10.8] | 4.3 [3.5–5.2] | 5.0 [4.0–6.0] |
| 2020 | 7.6 [6.1–9.1] | 4.2 [3.4–5.0] | 3.4 [2.4–4.3] |
| 2021 | 4.9 [3.8–5.9] | 3.0 [2.3–3.8] | 1.8 [1.3–2.3] |
| 2022 | 6.4 [5.3–7.5] | 4.0 [3.2–4.8] | 2.5 [1.8–3.2] |
| 2023 | 3.9 [2.8–5.1] | 2.4 [1.5–3.4] | 1.5 [1.0–2.0] |
|  |  |  |  |

CI, confidence interval.

^1^ Use on ≥20 of the past 30 days.

**Table 2.** Odds of frequent e-cigarette use (≥20 of the past 30 days) among US high-school students, 2014 to 2023

|  | **OR [95% CI] reporting frequent past-30-day use^1^ of…** | | |
| --- | --- | --- | --- |
| **Year** | **E-cigarettes** | **E-cigarettes only** | **E-cigarettes alongside other nicotine products** |
|  |  |  |  |
| 2014 | Ref | Ref | Ref |
| 2015 | 1.19 [0.80–1.77] | 1.88 [0.94–3.77] | 1.08 [0.71–1.64] |
| 2016 | 0.94 [0.65–1.38] | 1.33 [0.66–2.71] | 0.88 [0.59–1.30] |
| 2017 | 1.19 [0.76–1.87] | 2.11 [1.05–4.23] | 1.04 [0.64–1.67] |
| 2018 | 2.97 [2.09–4.22] | 7.02 [3.68–13.39] | 2.24 [1.56–3.21] |
| 2019 | 4.91 [3.45–6.99] | 15.64 [8.44–28.97] | 2.93 [2.01–4.25] |
| 2020 | 3.91 [2.69–5.69] | 15.16 [8.19–28.07] | 1.94 [1.28–2.95] |
| 2021 | 2.43 [1.67–3.56] | 10.78 [5.72–20.32] | 1.03 [0.68–1.57] |
| 2022 | 3.28 [2.30–4.69] | 14.25 [7.67–26.47] | 1.41 [0.92–2.15] |
| 2023 | 1.94 [1.27–2.97] | 8.63 [4.30–17.32] | 0.83 [0.53–1.30] |
|  |  |  |  |

CI, confidence interval; OR, odds ratio.

Data shown are the odds of frequent e-cigarette use in each year, relative to 2014. Each column shows results from a separate logistic regression model.

^1^ Use on ≥20 of the past 30 days.
