# Supplementary material for: Changing patterns of nicotine product use and nicotine dependence among United States high‐school students: The National Youth Tobacco Survey, 2014–2023
Source: Addiction. 2025 Jun 25;120(11):2215–22. doi: 10.1111/add.70120 (PMC12529234; doi:10.1111/add.70120)
Supplement: Supplementary file 4 — Data S4. Supplementary Material. [file ADD-120-2215-s004.docx]

**Table 1.** Cross-tabulation of past-30-day e-cigarette use and lifetime cigarette use among US high-school students in 2023

|  | **Number of lifetime cigarettes** | | | | | | | |
| --- | --- | --- | --- | --- | --- | --- | --- | --- |
| **Past-30-day  e-cigarette use** | Never smoked cigarettes, not even a puff | 1 or more puffs, never a whole cigarette | 1 cigarette | 2-5 cigarettes | 6-15 cigarettes | 16-25 cigarettes | 26-99 cigarettes | 100+ cigarettes |
|  |  |  |  |  |  |  |  |  |
| Any (≥1 day) |  |  |  |  |  |  |  |  |
| Row % [95% CI] | 60.7 [55.7–65.6] | 10.4 [7.7–13.9] | 4.7 [2.7–8.2] | 9.1 [6.7–12.2] | 3.2 [1.8–5.4] | 2.4 [1.5–3.8] | 4.2 [2.8–6.3] | 5.3 [3.2–8.8] |
| Column % [95% CI] | 6.2 [5.2–7.3] | 32.0 [25.4–39.4] | 42.9 [30.0–56.9] | 38.8 [28.6–49.9] | 64.0 [51.0–75.3] | 48.9 [22.1–76.3] | 59.2 [39.3–76.5] | 72.0 [52.4–85.7] |
|  |  |  |  |  |  |  |  |  |
| Frequent (≥20 days) |  |  |  |  |  |  |  |  |
| Row % [95% CI] | 49.0 [36.8–61.3] | 9.6 [5.1–17.2] | 4.3 [2.8–6.4] | 8.8 [5.8–13.3] | 5.9 [3.2–10.8] | 3.2 [1.7–6.2] | 7.6 [4.8–12.0] | 11.6 [6.2–20.6] |
| Column % [95% CI] | 2.0 [1.2–3.2] | 11.7 [7.0–19.1] | 15.6 [8.6–26.6] | 15.1 [8.9–24.4] | 48.1 [38.9–57.4] | 26.5 [11.6–50.0] | 42.5 [27.5–59.0] | 62.8 [41.4–80.2] |
|  |  |  |  |  |  |  |  |  |

CI, confidence interval.
