# Supplementary material for: Changing patterns of nicotine product use and nicotine dependence among United States high‐school students: The National Youth Tobacco Survey, 2014–2023
Source: Addiction. 2025 Jun 25;120(11):2215–22. doi: 10.1111/add.70120 (PMC12529234; doi:10.1111/add.70120)
Supplement: Supplementary file 5 — Data S5. Supplementary Material. [file ADD-120-2215-s003.docx]

**Table 1.** Past-30-day craving to use tobacco products among US high-school students, overall and by product use, 2014 to 2023

|  |  |  | **% [95% CI] reporting strong craving to use a tobacco product**  **in the past 30 days among those using…** | | | | | |  |
| --- | --- | --- | --- | --- | --- | --- | --- | --- | --- |
| **Year** | ***N*** | **E-cigarettes only** | | **Smokeless but no combustibles^1^** | **Combustibles but no cigarettes^2^** | **Cigarettes** | **Any nicotine product** | **Total population^3^** | |
|  |  |  | |  |  |  |  |  | |
| 2014 | 11,399 | 8.7 [5.3–12.0] | | 40.0 [32.8–47.2] | 17.4 [14.0–20.9] | 55.8 [51.6–59.9] | 31.9 [28.5–35.4] | 7.8 [6.6–9.0] | |
| 2015 | 9,433 | 9.1 [5.5–12.6] | | 42.8 [34.4–51.3] | 16.6 [13.4–19.8] | 48.9 [44.5–53.2] | 28.7 [25.3–32.2] | 7.2 [5.9–8.6] | |
| 2016 | 10,897 | 10.0 [6.9–13.1] | | 30.3 [22.3–38.4] | 19.6 [16.5–22.6] | 55.5 [49.4–61.6] | 32.3 [28.4–36.2] | 6.5 [5.3–7.8] | |
| 2017 | 10,186 | 8.4 [5.1–11.7] | | 30.1 [15.2–45.0] | 21.6 [15.3–27.9] | 46.3 [42.4–50.1] | 27.9 [24.1–31.8] | 5.5 [4.3–6.7] | |
| 2018 | 10,991 | 15.9 [12.7–19.0] | | 35.3 [26.9–43.7] | 22.5 [17.7–27.2] | 51.8 [47.7–56.0] | 29.3 [26.5–32.0] | 7.9 [6.8–9.0] | |
| 2019 | 10,097 | 15.8 [13.2–18.4] | | 29.4 [21.4–37.3] | 25.5 [21.0–30.0] | 52.2 [45.7–58.6] | 25.5 [22.0–29.0] | 8.0 [6.5–9.5] | |
| 2020 | 7,453 | 27.6 [24.2–31.1] | | 36.6 [25.8–47.4] | 30.5 [24.5–36.6] | 53.2 [44.9–61.5] | 33.9 [30.6–37.3] | 8.0 [6.5–9.5] | |
| 2021 | 10,515 | 20.3 [15.4–25.3] | | 27.4 [16.0–38.8] | 25.0 [18.1–31.9] | 39.7 [31.3–48.2] | 24.5 [20.9–28.2] | 3.3 [2.5–4.1] | |
| 2022 | 16,118 | 24.6 [20.6–28.5] | | 27.1 [19.7–34.4] | 18.3 [13.4–23.3] | 41.7 [32.1–51.2] | 25.7 [22.3–29.2] | 4.2 [3.4–5.0] | |
| 2023 | 10,879 | 16.9 [12.5–21.3] | | 18.5 [11.7–25.4] | 23.9 [13.8–33.9] | 30.2 [18.2–42.2] | 20.2 [16.4–24.0] | 2.5 [1.9–3.1] | |
|  |  |  | |  |  |  |  |  | |

CI, confidence interval.

^1^ Includes chewing tobacco, snuff, or dip; snus; heated tobacco products; nicotine pouches; other oral nicotine products.

^2^ Includes cigars, cigarillos, or little cigars; pipes filled with tobacco; bidis; tobacco in a hookah or waterpipe.

^3^ Includes participants reporting no past-30-day use of any nicotine product.

**Table 2.** Odds of past-30-day craving to use tobacco products among US high-school students, overall and by product use, 2014 to 2023

|  |  | **OR [95% CI] of reporting strong craving to use a tobacco product**  **in the past 30 days among those using…** | | | | |
| --- | --- | --- | --- | --- | --- | --- |
| **Year** | **E-cigarettes only** | **Smokeless but no combustibles^1^** | **Combustibles but no cigarettes^2^** | **Cigarettes** | **Any nicotine product** | **Total population^3^** |
|  |  |  |  |  |  |  |
| 2014 | Ref | Ref | Ref | Ref | Ref | Ref |
| 2015 | 1.05 [0.59–1.87] | 1.12 [0.72–1.75] | 0.94 [0.68–1.30] | 0.76 [0.60–0.96] | 0.86 [0.69–1.08] | 0.92 [0.72–1.18] |
| 2016 | 1.18 [0.69–2.00] | 0.65 [0.41–1.05] | 1.15 [0.85–1.56] | 0.99 [0.73–1.33] | 1.02 [0.80–1.29] | 0.82 [0.63–1.07] |
| 2017 | 0.96 [0.54–1.74] | 0.65 [0.31–1.36] | 1.30 [0.84–2.01] | 0.68 [0.55–0.85] | 0.83 [0.65–1.06] | 0.68 [0.51–0.90] |
| 2018 | 1.99 [1.24–3.19] | 0.82 [0.52–1.30] | 1.37 [0.96–1.97] | 0.85 [0.68–1.08] | 0.88 [0.72–1.08] | 1.01 [0.81–1.26] |
| 2019 | 1.98 [1.26–3.11] | 0.62 [0.39–1.00] | 1.62 [1.16–2.27] | 0.86 [0.64–1.17] | 0.73 [0.57–0.93] | 1.02 [0.79–1.33] |
| 2020 | 4.03 [2.58–6.28] | 0.87 [0.51–1.48] | 2.08 [1.44–3.00] | 0.90 [0.63–1.30] | 1.09 [0.88–1.36] | 1.02 [0.79–1.32] |
| 2021 | 2.70 [1.62–4.48] | 0.57 [0.30–1.06] | 1.58 [1.02–2.43] | 0.52 [0.36–0.77] | 0.69 [0.54–0.89] | 0.40 [0.29–0.54] |
| 2022 | 3.44 [2.17–5.46] | 0.56 [0.35–0.89] | 1.06 [0.71–1.60] | 0.57 [0.37–0.87] | 0.74 [0.58–0.94] | 0.52 [0.40–0.67] |
| 2023 | 2.15 [1.29–3.57] | 0.34 [0.20–0.58] | 1.48 [0.83–2.66] | 0.34 [0.19–0.61] | 0.54 [0.41–0.71] | 0.31 [0.23–0.41] |
|  |  |  |  |  |  |  |

CI, confidence interval; OR, odds ratio.

^1^ Includes chewing tobacco, snuff, or dip; snus; heated tobacco products; nicotine pouches; other oral nicotine products.

^2^ Includes cigars, cigarillos, or little cigars; pipes filled with tobacco; bidis; tobacco in a hookah or waterpipe.

^3^ Includes participants reporting no past-30-day use of any nicotine product.

**Table 3.** Wanting to use a tobacco product within 30 minutes of waking among US high-school students, overall and by product use, 2014 to 2023

|  |  |  | **% [95% CI] reporting wanting to use a tobacco product**  **within 30 minutes of waking among those using…** | | | | | |  |
| --- | --- | --- | --- | --- | --- | --- | --- | --- | --- |
| **Year** | ***N*** | **E-cigarettes only** | | **Smokeless but no combustibles^1^** | **Combustibles but no cigarettes^2^** | **Cigarettes** | **Any nicotine product** | **Total population^3^** | |
|  |  |  | |  |  |  |  |  | |
| 2014 | 11,399 | 1.0 [0.0–1.9] | | 12.4 [8.0–16.7] | 4.7 [2.9–6.4] | 26.7 [22.7–30.7] | 12.8 [10.5–15.1] | 3.3 [2.6–3.9] | |
| 2015 | 9,433 | 2.0 [0.6–3.5] | | 18.7 [10.5–26.9] | 4.3 [2.5–6.0] | 25.7 [21.4–30.0] | 12.7 [10.2–15.2] | 3.3 [2.5–4.1] | |
| 2016 | 10,897 | 1.7 [0.4–3.0] | | 14.5 [7.2–21.9] | 7.5 [5.2–9.9] | 31.1 [25.8–36.4] | 16.0 [12.7–19.2] | 3.4 [2.5–4.3] | |
| 2017 | 10,186 | 2.9 [0.8–5.0] | | 12.4 [5.2–19.5] | 9.8 [5.7–13.9] | 24.4 [19.8–29.0] | 13.6 [10.9–16.3] | 2.8 [2.1–3.6] | |
| 2018 | 10,991 | 7.2 [4.8–9.6] | | 15.5 [9.4–21.6] | 10.8 [7.0–14.7] | 29.1 [25.2–33.0] | 15.0 [13.0–17.1] | 4.2 [3.5–4.9] | |
| 2019 | 10,097 | 8.7 [6.9–10.5] | | 20.8 [13.3–28.3] | 15.3 [10.9–19.7] | 32.8 [27.7–38.0] | 15.4 [12.7–18.0] | 5.4 [3.8–7.0] | |
| 2020 | 7,453 | 15.9 [13.2–18.6] | | 19.2 [9.8–28.6] | 16.2 [11.3–21.2] | 31.7 [21.5–42.0] | 19.3 [16.9–21.8] | 4.6 [3.7–5.5] | |
| 2021 | 10,515 | 17.5 [13.8–21.1] | | 21.9 [11.9–31.8] | 15.2 [8.9–21.5] | 33.0 [23.6–42.4] | 19.6 [16.7–22.5] | 2.6 [2.0–3.2] | |
| 2022 | 16,118 | 19.2 [16.0–22.4] | | 24.2 [16.1–32.2] | 17.1 [12.0–22.1] | 33.7 [25.3–42.1] | 21.0 [18.1–24.0] | 3.5 [2.8–4.1] | |
| 2023 | 10,879 | 13.9 [9.4–18.5] | | 10.3 [4.8–15.7] | 15.3 [8.7–21.8] | 29.9 [16.9–42.9] | 15.9 [12.0–19.9] | 2.0 [1.4–2.6] | |
|  |  |  | |  |  |  |  |  | |

CI, confidence interval.

^1^ Includes chewing tobacco, snuff, or dip; snus; heated tobacco products; nicotine pouches; other oral nicotine products.

^2^ Includes cigars, cigarillos, or little cigars; pipes filled with tobacco; bidis; tobacco in a hookah or waterpipe.

^3^ Includes participants reporting no past-30-day use of any nicotine product.

**Table 4.** Odds of wanting to use a tobacco product within 30 minutes of waking among US high-school students, overall and by product use, 2014 to 2023

|  |  | **OR [95% CI] of reporting wanting to use a tobacco product**  **within 30 minutes of waking among those using …** | | | | | | |
| --- | --- | --- | --- | --- | --- | --- | --- | --- |
| **Year** | **E-cigarettes only** | | **Smokeless but no combustibles^1^** | **Combustibles but no cigarettes^2^** | **Cigarettes** | **Any nicotine product** | **Total population^3^** |  |
|  |  | |  |  |  |  |  |  |
| 2014 | Ref | | Ref | Ref | Ref | Ref | Ref |  |
| 2015 | 2.17 [0.69–6.81] | | 1.63 [0.85–3.11] | 0.91 [0.52–1.59] | 0.95 [0.70–1.29] | 0.99 [0.74–1.34] | 1.01 [0.74–1.40] |  |
| 2016 | 1.76 [0.52–5.94] | | 1.20 [0.60–2.40] | 1.66 [1.00–2.76] | 1.24 [0.90–1.70] | 1.30 [0.95–1.78] | 1.03 [0.74–1.45] |  |
| 2017 | 3.11 [0.94–10.27] | | 1.00 [0.47–2.11] | 2.21 [1.23–3.98] | 0.89 [0.65–1.22] | 1.08 [0.80–1.46] | 0.86 [0.62–1.21] |  |
| 2018 | 8.08 [2.97–21.96] | | 1.30 [0.72–2.36] | 2.48 [1.45–4.25] | 1.13 [0.86–1.48] | 1.21 [0.93–1.56] | 1.30 [1.00–1.70] |  |
| 2019 | 9.89 [3.79–25.86] | | 1.86 [1.03–3.36] | 3.68 [2.22–6.10] | 1.34 [0.99–1.82] | 1.24 [0.93–1.65] | 1.67 [1.15–2.44] |  |
| 2020 | 19.76 [7.59–51.42] | | 1.68 [0.84–3.38] | 3.96 [2.35–6.65] | 1.28 [0.77–2.10] | 1.64 [1.27–2.11] | 1.41 [1.06–1.89] |  |
| 2021 | 22.07 [8.37–58.20] | | 1.98 [1.00–3.93] | 3.65 [1.99–6.72] | 1.35 [0.85–2.14] | 1.66 [1.27–2.18] | 0.79 [0.58–1.09] |  |
| 2022 | 24.77 [9.51–64.53] | | 2.26 [1.26–4.05] | 4.20 [2.50–7.06] | 1.40 [0.91–2.13] | 1.82 [1.39–2.38] | 1.06 [0.79–1.41] |  |
| 2023 | 16.85 [6.17–46.03] | | 0.81 [0.41–1.61] | 3.67 [1.98–6.80] | 1.17 [0.63–2.20] | 1.29 [0.91–1.83] | 0.60 [0.42–0.85] |  |
|  |  | |  |  |  |  |  |  |

CI, confidence interval; OR, odds ratio.

^1^ Includes chewing tobacco, snuff, or dip; snus; heated tobacco products; nicotine pouches; other oral nicotine products.

^2^ Includes cigars, cigarillos, or little cigars; pipes filled with tobacco; bidis; tobacco in a hookah or waterpipe.

^3^ Includes participants reporting no past-30-day use of any nicotine product.


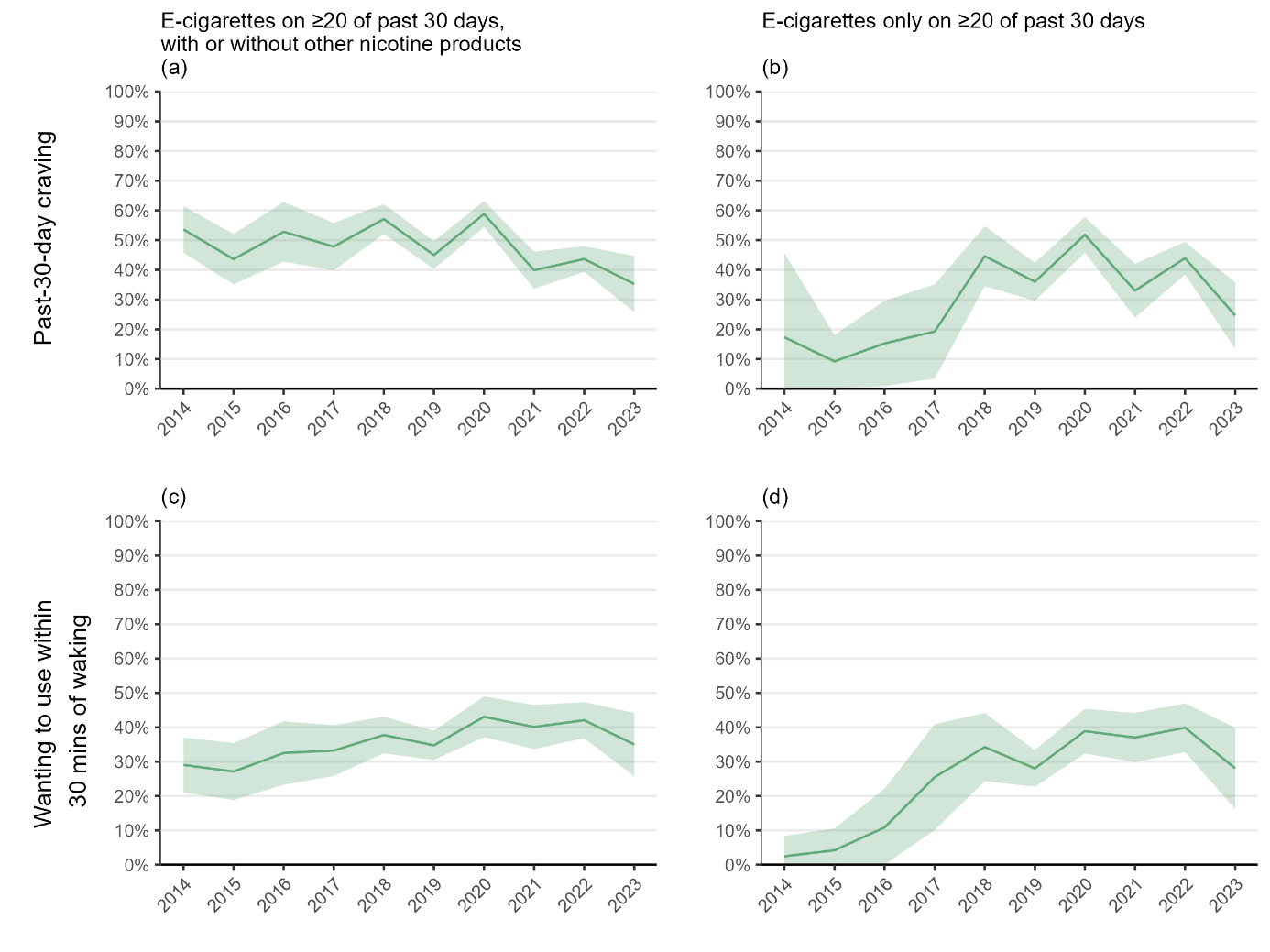


**Figure 1. Nicotine dependence among US high-school students reporting frequent e-cigarette use, 2014 to 2023.** Data shown are estimates of the proportions (with 95% confidence intervals) of participants using e-cigarettes on ≥20 of the past 30 days who reported symptoms of nicotine dependence.
